# The role of pancreas to improve hyperglycemia in STZ-induced diabetic rats by thiamine disulfide

**DOI:** 10.1038/s41387-022-00211-5

**Published:** 2022-06-20

**Authors:** Mahtab Ghanbari Rad, Mohammadreza Sharifi, Rokhsareh Meamar, Nepton Soltani

**Affiliations:** 1 Department of Physiology, School of Medicine, Isfahan University of Medical Sciences, Isfahan, Iran; 2 Department of Genetics and Molecular Biology, School of Medicine, Isfahan University of Medical Sciences, Isfahan, Iran; 3 Department of Clinical Toxicology, School of Medicine, Isfahan University of Medical Sciences, Isfahan, Iran

**Keywords:** Type 1 diabetes, Metabolism

## Abstract

**Background:**

The present study investigated the effect of thiamine disulfide (TD) on the pancreas in terms of hyperglycemia improvement and insulin sensitivity increase in diabetic male rats. We also aimed to study the function of *Pdx1* (pancreatic and duodenal homeobox 1) and *Glut2* (glucose transporter 2) genes in pancreatic tissue.

**Methods:**

Type 1 diabetes was induced through injection of 60 mg/kg streptozotocin (STZ). The diabetic rats were divided into four groups, namely diabetic control (DC), diabetic treated with thiamine disulfide (D-TD), diabetic treated with insulin (D-insulin), and diabetic treated with TD and insulin (D-insulin+TD). The non-diabetic (NDC) and diabetic groups received a normal diet (14 weeks). Blood glucose level and body weight were measured weekly; insulin tolerance test (ITT) and glucagon tolerance test (GTT) were performed in the last month of the study. The level of serum insulin and glucagon were measured monthly and a hyperglycemic clamp (Insulin Infusion rate (IIR)) was done for all the groups. Pancreas tissue was isolated so that *Pdx1*and *Glut2* genes expression could be measured.

**Results:**

We observed that TD therapy decreased blood glucose level, ITT, and serum glucagon levels in comparison with those of the DC group; it also increased serum insulin levels, IIR, and expression of *Pdx1* and *Glut2* genes in comparison with those of the DC group.

**Conclusion:**

Administration of TD could improve hyperglycemia in type 1 diabetic animals through improved pancreas function. Therefore, not only does TD have a significant effect on controlling and reducing hyperglycemia in diabetes, but it also has the potential to decrease the dose of insulin administration.

## Introduction

Diabetes is a serious lifelong disease, always characterized by abnormally high blood glucose levels due to insulin production disorder or decreased insulin sensitivity and function [1]. Type 1 diabetes (T1D) is an autoimmune disorder that leads to the destruction of pancreatic β-cells and occurs at an early age [2, 3]. The pancreas plays an important role in regulating blood glucose levels by secreting insulin and glucagon hormones. There is a direct link between diabetes and pancreatic damage (impaired insulin secretion) [4, 5]. Blood glucose levels begin to rise over time once affected by impaired insulin function. If insulin resistance develops, the effectiveness of insulin decreases; [6, 7] insulin resistance (IR) is a dynamic pathological disorder resulting from inadequate cellular response to insulin [8, 9] in insulin-dependent cells, which occurs in various metabolic disorders, including type 2 diabetes (T2D) and metabolic syndrome [10, 11]. Insulin resistance has also been suggested to occur in T1D. Previously, intensive insulin therapy was applied in T1DM in order to keep the glucose level as close to normal as possible and prevent hypoglycemia [3, 12]; meanwhile, studies have shown that long-term insulin administration leads to insulin resistance and exacerbates the complications of diabetes due to decreased insulin receptor regulation [13, 14]. Clinical and experimental evidence has suggested that patients with insulin resistance in T1D may have abnormal glucagon action [3, 15]. Thiamine or vitamin B1 is a coenzyme involved in the metabolism of sugars; [16] it is essential for the synthesis and secretion of insulin, and its level decreases in diabetes [17, 18]. In thiamine deficiency, glucose is metabolized through metabolic pathways that can stimulate insulin resistance and the complications of diabetes [19, 20]. Previous studies have reported that taking thiamine supplements can improve diabetes [21, 22]. In addition, Glut2 is a glucose transporter in pancreatic β-cells and its inactivation leads to impaired insulin secretion [23, 24]. Homeobox 1 and duodenal transcription factor (Pdx1) play an essential role in the maintenance and survival of pancreatic cells [25]. Pdx1 is vital for the pancreatic β-cells differentiation [26, 27] and maintains the function of β-cells by regulating the genes involved in glucose homeostasis, such as insulin, glucose transporter 2 (Glut2), and glucokinase (GK). Decreased expression of this gene causes a lack of response to glucose, decreased glucose-stimulated insulin secretion, and increased β-cells apoptosis and diabetes [28].

Today, numerous thiamine compounds have been artificially innovated, which due to their biochemical structure, have better and more desirable absorption and effectiveness than free thiamine, such as sulbutiamine or TD; in this combination, the two free thiamine are mixed using a disulfide bond and structural modification [29, 30]. Unlike thiamine, the solubility of TD in fat is higher than that of water, which facilitates its absorption and has a good function in sugar metabolism [31].

Considering the fact that insulin resistance developed after prolonged exogenous insulin intake in T1D patients as well as the complications of thiamine deficiency in these patients, we evaluated the effect of TD on the improvement of blood glucose levels, pancreas function, and insulin sensitivity in STZ-induced diabetic rats. Insulin sensitivity was assessed with hyperglycemic-euinsulinemic clamp technique and pancreatic gene expression of *Glut2* and *Pdx1*.

## Materials and methods

### Animals

The animals were utilized according to the criteria mentioned in (NIH No. 85 # 23, amended in 1985). The local animal ethics permission approved this work under the code IR. MUI.MED.REC.1398.572. Herein, 50 male Wistar rats, aged 4 weeks, were kept in the weight range of 180–250 g for 14 weeks at room temperature (22 ± 20 °C) and relative humidity of 50 ± 5% with 12:12 hours of dark and light control cycles. The appropriately classified rats were kept in special cages with free access to water and food.

### Diabetes induction

Diabetes was induced through intraperitoneal (IP) injection with a single dose (60 mg/kg) of STZ (Sigma-Aldrich Inc., USA) [32]. One week later, their blood glucose levels were determined via a glucometer (ACCU-CHEK Active, Germany), and the animals with blood glucose levels above 250 mg/dl were considered diabetic [32]. The animals were randomly divided into five groups (n = 7): 1. control intact or non-diabetic group (NDC); 2. diabetic control (DC); 3. diabetic treated with insulin (2.5 U/kg, BID (1/3 in the morning and 2/3 in the evening)) (D-insulin); 4. diabetic treated with TD (40 mg/kg/day, IP, was obtained based on the doses of the pilot study) (D-TD); 5. diabetic treated with TD (40 mg/kg) and insulin (2.5 U/kg/day) (D-insulin+TD). All the diabetic and NDC groups were studied for 14 weeks under a normal diet and with free access to water. All the animal-involved procedures in this research were in line with the standards of the local ethical committee.

### Weekly blood glucose levels and body weight

Bodyweight and blood glucose levels were monitored on a weekly basis before and after STZ injection in all the groups. The rats were weighed using a digital scale. Their blood glucose levels were recorded with a glucometer from the tail vein [33].

### Glucagon tolerance test (GTT)

At the end of the treatment period (after 14 weeks), a glucagon tolerance test was done on the fasting animals. After recording the fasting blood glucose, glucagon was injected (20 μg/kg, IP) and tail vein blood glucose was measured at 0, 20, 30, 40, 60, 90, and 120 minutes [34].

### Intraperitoneal insulin tolerance test (ITT)

The insulin tolerance test (ITT), an index of peripheral utilization of glucose and insulin resistance, was performed in the last month following the treatment. All the groups received regular insulin (2.5 U/kg, IP) and blood glucose was measured at 0, 20, 30, 40, 60, 90, and 120 minutes. The results were expressed as an integrated area under the curve of glucose (AUC glucose) [33].

### Biochemical analysis

Monthly tail vein blood sampling was performed in all the groups under anesthesia; the serum was separated for biochemical analysis. Serum insulin and glucagon were assessed according to ELISA kit instructions (Zell Bio GmbH, Germany) [33].

### Surgery

The animals were anesthetized (100 mg/kg of ketamine and 8 mg/kg of xylazine, IP) [35], and common carotid artery and jugular vein were cannulated by 50 heparinized polyethylene tubes and then fixed to the back of the animal’s neck. After this operation, the animals were monitored for 3–5 days [33].

### Hyperglycemic-euinsulinemic clamp

After recovery, the animals were fasted for 12 hours. After weighing, the carotid artery and jugular vein cannula were connected to two microinjection pumps (New Era Pump System Inc. Farmingdale, New York, USA) that delivered insulin and glucose simultaneously. Slow injection through the Y interface and carotid artery was carried out for blood sampling. In this method, constant amounts of 25% glucose and a variable amount of insulin (20 mu/kg/min) were injected for 5 hours. Blood glucose level was checked every 10 minutes through a glucometer, and in the last half hour, it was recorded to be in the range of 95–100 mg/dl. In addition, to calculate the sensitivity of the whole body to insulin, the amount of insulin injected in the last 30 minutes of the clamp on top of the amount of blood glucose levels in this range was measured [35].

### Pancreas tissue preparation and real-time PCR

The pancreatic tissue was forthwith frozen in liquid nitrogen and stored at −80 °C for future measurements of gene expression of *Pdx1* and *Glut2*. We utilized 5 μl of extracted RNA (according to the protocol, Anacell, lot N: CS0021) for the synthesis of cDNA via Reverse Transcriptase (RT) according to the kit instruction (Anacell, lot N: CS0021). The real-time PCR technique was performed using the SYBR-green method (Biosystems Applied); 1 μl of total cDNA was mixed with 10 microliters of 2×SYBR Green PCR mixed with ROX, treated with water, and 10 pmol/ml of each of the sensory and antisense primers (Table 1) for the measured genes. Mean beta-actin expression was used as an internal reference gene to normalize the input cDNA. Finally, the recorded CTs were examined to study the expression of the genes [35, 36].

**Table 1:** Primers for quantitative real-time PCR analysis of gene expression.

Gene	Primer(R: reveres, F: forward)	Reference
Beta-actin	R: CTGACCCATACCCACCATCAC	Designed with NCBI’s Primer-BLAST
	F: ACAACCTTCTTGCAGCTCCTC	
Pdx1	R: TGTAGGCTGTACGGGTCCTC	Designed with NCBI’s Primer-BLAST
	F: CCCGAATGGAACCGAGACTG	
Glut2	R: GAACTGGAAGGAACCCAGCA	Designed with NCBI’s Primer-BLAST
	F: GCAACATGTCAGAAGACAAGATCA	

### Statistical analysis

The obtained data are expressed as mean ± SEM. Kolmogorov–Smirnov test was used to check the normality of all the variables. The comparisons among the groups were studied with two-way analysis of variance followed by Tukey test, using SPSS software; *P* < 0.05 was considered to be significant.

## Results

### Effect of TD on blood glucose levels and body weight

**Fig. 1:**
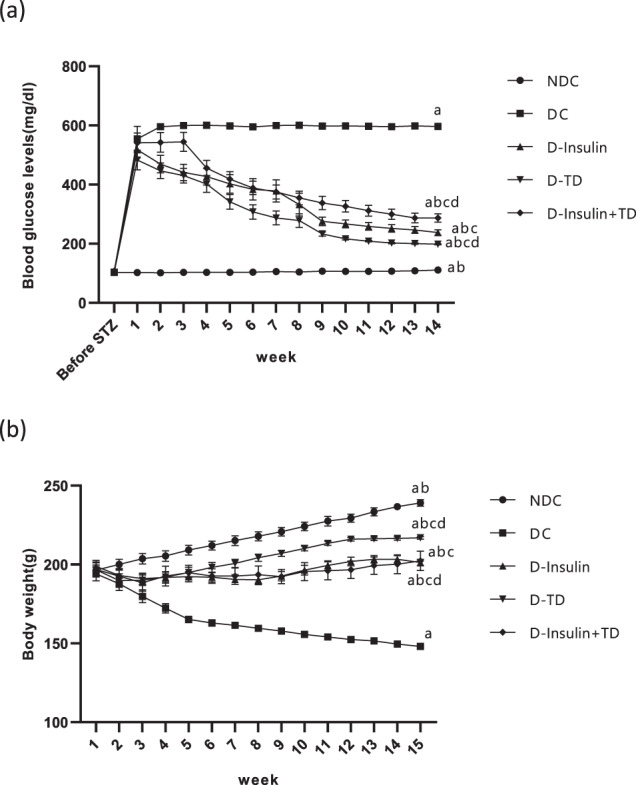
Effect of TD on blood glucose levels and body weight in male rats. Comparison of fed blood glucose levels (**a**) and body weight (**b**) in the non-diabetic control (NDC), diabetic control (DC), D-insulin, D-TD, and D-insulin+TD groups. DC group received 60 mg/kg STZ. D-insulin group diabetic animals were treated with 2.5 U/kg insulin twice per day (1/3 morning and 2/3 in the evening), D-TD group diabetic animals were treated with daily IP injection of 40 mg/kg thiamine disulfide, D-insulin+TD group diabetic animals treated with TD and insulin (2.5 U/kg insulin +40 mg/kg TD once per day). Data are expressed as mean ± S.E.M (*N* = 7). **a** Significant difference in blood glucose and body weight. between the DC group and other groups (DC vs NDC (*p* < 0.0001), DC vs D-insulin and D-insulin+TD (*p* < 0.01), DC vs D-TD (*p* < 0.001). **b** Significant difference in blood glucose levels and body weight between NDC group and other groups (NDC vs D-TD (*p* < 0.01), NDC vs D-insulin+TD (*p* < 0.001). **c** Significant difference between D-insulin group and the other two treatment groups (D-TD and D-insulin+TD), (*p* < 0.01) for blood glucose levels and (*p* < 0.001) for body weight. **d** Significant difference in blood glucose levels and body weight between D-TD and D-insulin+TD groups (*p* < 0.001).

Changes in blood glucose levels were measured in all groups. Induction of diabetes significantly increased (*p* < 0.0001) blood glucose level in compare with NDC group (NDC: 102.5 ± 1.2 mg/dl, DC: 554.2 ± 42.4 mg/dl, Fig. 1a). Hyperglycemia in the animals continued throughout the study. Compared to the DC group, the blood glucose levels in all treatment groups for 14 weeks were significantly reduced (*p* < 0.0001) (Fig. 1a). The D-TD group showed a greater improvement in glucose reduction than the other treatment groups (D-insulin and D-insulin +TD). (D-TD: 198.44 ± 1.8 mg/dl, D-insulin: 237.5 ± 9.1 mg/dl, D-insulin +TD: 287.16 ± 14.1 mg/dl).

Body weight was measured weekly and the results showed that induction of diabetes significantly decreased (*p* < 0.0001) body weight compared to the NDC group and this continued until the end of the study (NDC: 200 ± 3.2 g DC: 187.81 ± 4.22 g), (Fig. 1b). In all treatment groups, the animal’s weight significantly increased (*p* < 0.0001) in comparison to the DC group (Fig. 1b). Weight gain in the D-TD group was more than in the other treatment groups (D-insulin and D-insulin +TD) (Fig. 1b). Among treatment groups, there was a significant difference (*p* < 0.001) concerning the body weight (D-TD: 216.88 ± .78 g, D-insulin: 201.2 ± 2.07 g, D-insulin +TD: 202.33 ± 6.14 g).

### Effect of TD on glucagon tolerance test (GTT)

**Fig. 2:**
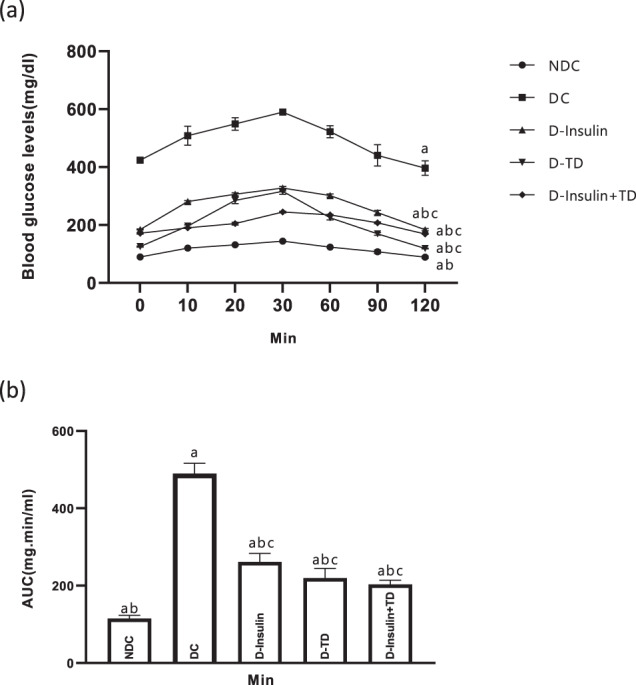
Effect of TD on changes in blood glucose levels in the glucagon tolerance test (GTT) of male rats. Comparison of last month’s glucagon tolerance test (GTT) (**a**) and the area under the glycemic curve (AUC) (**b**) in the non-diabetic control (NDC), diabetic control (DC), D-insulin, D-TD, and D-insulin+TD groups. DC group received 60 mg/kg STZ. D-insulin group diabetic animals were treated with 2.5 U/kg insulin twice per day (1/3 morning and 2/3 in the evening), the D-TD group diabetic animals were treated with daily IP injection of 40 mg/kg thiamine disulfide, D-insulin+TD group diabetic animals treated with TD and insulin (2.5 U/kg insulin +40 mg/kg TD once per day). Data are expressed as mean ± S.E.M (*N* = 7). **a** Significant difference in AUC and GTT between the DC group and other groups (DC vs NDC (*p* < 0.0001), DC vs other treatments (*p* < 0.001). **b** Significant difference in AUC and GTT between the NDC group and other groups (*p* < 0.001). **c** Significant difference in AUC and GTT between the D-insulin group and the other two treatments (D-TD and D-insulin+TD) groups (*p* < 0.001).

In the last month of treatment, a glucagon tolerance test was performed in all groups. In the DC group, the area under the glycemic curve (AUC) was higher than the NDC animals (*p* < 0.0001; Fig. 2a, b). The AUC significantly decreased in all treatment groups (D-insulin vs D-TD *p* < 0.001, D-insulin vs D-insulin +TD, *p* < 0.001, Fig. 2a, b). The reduction was more effective (*p* < 0.001) in the D-TD group than other treatment (D-insulin and D-insulin+TD) groups. Also, all treatment groups showed a significantly positive difference in comparison to the NDC group (*p* < 0.0001; Fig. 2b). (NDC: 14123.75 ± 297.29 mg.min/ml DC: 59306.25 ± 2353.24 mg.min/ml D-insulin: 31960 ± 483.58 mg.min/ml D-TD: 25210 ± 318.44 mg.min/ml D-insulin +TD: 25388.75 ± 148.58 mg.min/ml).

### Effect of TD on (ITT)

**Fig. 3:**
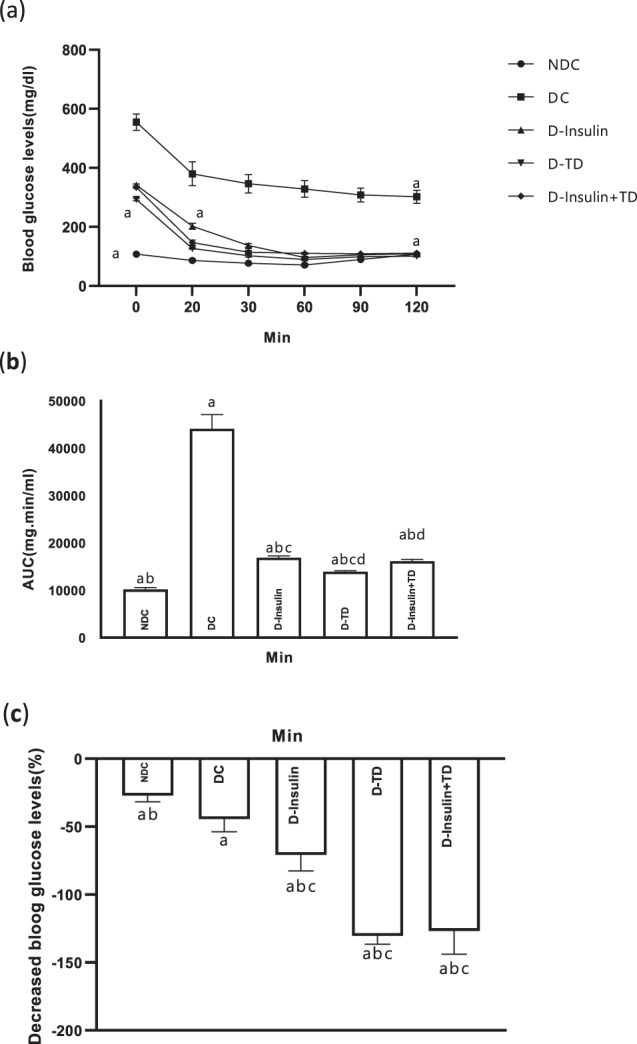
Effect of TD on changes in blood glucose levels in the insulin tolerance test (ITT) of male rats. Comparison of the insulin tolerance test (ITT) in the last month (**a**) and the area under the curve (AUC) (**b**) and decreased blood glucose level (**c**) in the non-diabetic control (NDC), diabetic control (DC), D-insulin, D-TD, and D-insulin+TD groups. DC group received 60 mg/kg STZ. The D-insulin group diabetic animals were treated with 2.5 U/kg insulin twice per day (1/3 morning and 2/3 in the evening), D-TD group diabetic animals were treated with daily IP injection of 40 mg/kg thiamine disulfide, D-insulin+TD group diabetic animals treated with TD and insulin (2.5 U/kg insulin +40 mg/kg TD once per day). Data are expressed as mean ± S.E.M (*N* = 7). **a** Significant difference in ITT and AUC between the DC group and other groups (*p* < 0.0001) and significant difference in decreased blood glucose level (DC vs NDC (*p* < 0.05), DC vs D-insulin (*p* < 0.01), DC vs D-TD and D-insulin+TD (*p* < 0.001). **b** Significant difference in AUC between the NDC group and other groups (*p* < 0.0001) and significant difference in decreased blood glucose level (NDC vs D-insulin (*p* < 0.01), NDC vs D-TD and D-insulin+TD (*p* < 0.0001). **c** Significant difference between the D-insulin group and the other two treatments (D-TD and D-insulin+TD) groups (*p* < 0.01) for AUC and (*p* < 0.001) for a decreased blood glucose level. d Significant difference in AUC between the D-TD and D-insulin+TD groups (*p* < 0.01).

At the end of the study, ITT was performed for all animals. The level of AUC in the DC group was higher than the NDC group and all treatment groups (*p* < 0.0001; Fig. 3a, b). But the AUC did not reach the NDC level in all treatment groups (Fig. 3b, c). There was not a significant difference between the two groups of D-insulin and D-insulin +TD (Fig. 3c). (NDC: 10172.5 ± 442.82 mg.min/ml DC: 44086.25 ± 3027.47 mg.min/ml D-insulin: 16908.75 ± 356.85 mg.min/ml D-TD: 13927.5 ± 226.66 mg.min/ml D-insulin +TD: 16161.25 ± 362.52 mg.min/ml).

### Effect of TD in the IIR

**Fig. 4:**
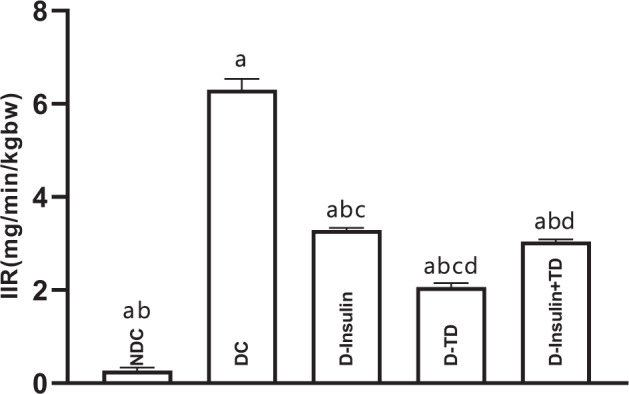
Effect of TD on insulin sensitivity in male rats. Comparison of IIR in the non-diabetic control (NDC), diabetic control (DC), D-insulin, D-TD, and D-insulin+TD groups. DC group received 60 mg/kg STZ. D-insulin group diabetic animals were treated with 2.5 U/kg insulin twice per day (1/3 morning and 2/3 in the evening), D-TD group diabetic animals were treated with daily IP injection of 40 mg/kg thiamine disulfide, D-insulin+TD group diabetic animals were treated with TD and insulin (2.5 U/kg insulin +40 mg/kg TD once per day). Data are expressed as mean ± S.E.M (*N* = 7). **a** Significant difference in IIR between the DC group and other groups (DC vs NDC (*p* < 0.00001), DC vs D-insulin and D-insulin+TD (*p* < 0.001), DC vs D-TD (*p* < 0.0001). **b** Significant difference in IIR between the NDC group and other groups (NDC vs D-insulin and D-insulin+TD (*p* < 0.01), NDC vs D-TD (*p* < 0.001)). **c** Significant difference in IIR between the D-insulin group and the other two treatments (D-insulin vs D-TD groups (*p* < 0.0001). **d** Significant difference in IIR between the D-TD and D-insulin+TD groups (*p* < 0.0001).

After 14 weeks of treatment, a hyperglycemic-euinsulinemic clamp test was performed to assess whole-body insulin sensitivity in all animals. In this type of test, the blood glucose level was clamped at 100 ± 5 mg/dl. TD therapy significantly increased (*p* < 0.0001) the rate of insulin injection (IIR) required to maintain euglycemia during the injection of constant glucose rate in comparison with the DC group (Fig. 4). IIR was lower in D-TD group rats than in animals in the D-insulin and D-insulin +TD groups. (*p* < 0.001, Fig. 4). In all treatment groups, the rate of IIR was higher than in the NDC group (*p* < 0.0001; Fig. 4). (NDC: 0.2678 ± 0.06 μ/min/kgbw DC: 6.3027 ± 0.23 μ/min/kgbw D-insulin: 3.2905 ± 0.04 μ/min/kgbw D-TD: 2.0603 ± 0.08 μ/min/kgbw D-insulin +TD: 3.040 ± 0.05 μ/min/kgbw).

### Changes in serum insulin and glucagon levels

**Fig. 5:**
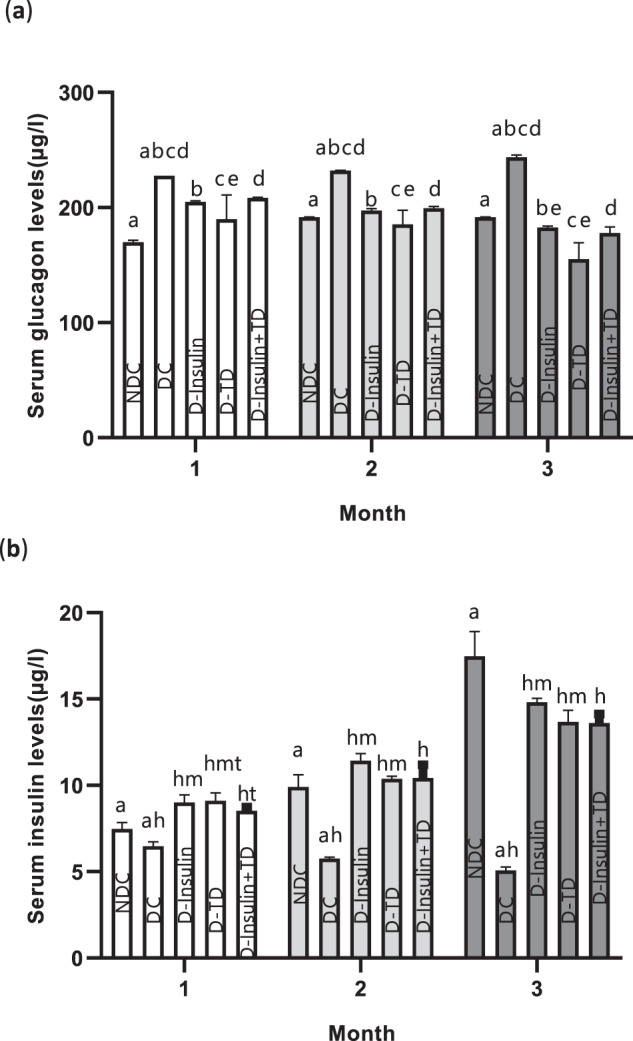
Effect of TD on changes in serum insulin and glucagon levels in male rats. Comparison of serum glucagon levels (**a**) and serum insulin levels (**b**) over three months in the non-diabetic control (NDC), diabetic control (DC), D-insulin, D-TD, and D-insulin+TD groups. The DC group received 60 mg/kg STZ. D-insulin treated with 2.5 U/kg insulin twice per day (1/3 morning and 2/3 in the evening), D-TD group treated with daily IP injection of 40 mg/kg thiamine disulfide, D-insulin+TD group treated with TD and insulin (2.5 U/kg insulin +40 mg/kg TD once per day). Data are expressed as mean ± S.E.M (*N* = 7). **a** Significant difference between NDC and DC groups (*p* < 0.0001) during the three months for serum glucagon levels. **b** Significant difference between the DC and D-insulin groups (first month: (*p* < 0.01), second month (*p* < 0.001), third month (*p* < 0.0001)) for serum glucagon levels. **c** Significant difference between the DC and D-TD groups (first month: (*p* < 0.01), second month (*p* < 0.001), third month (*p* < 0.0001)) for serum glucagon levels. **d** Significant difference between the DC and D-insulin+TD groups (first month: (*p* < 0.001), second month (*p* < 0.001), third month (*p* < 0.0001)) for serum glucagon levels. **e** Significant difference between the D-insulin and D-TD groups (*p* < 0.001) during the three months for serum glucagon levels. **f** Significant difference between the D-TD and D-insulin+TD groups (*p* < 0.001) during the three months for serum glucagon levels. **r** Significant difference between the NDC and DC groups (first month: (*p* < 0.01), second month (*p* < 0.001), third month (*p* < 0.0001)) for serum insulin levels. **h** Significant difference between the DC group and (D-insulin, D-TD and D-insulin+TD groups (first month: (*p* < 0.01), second month (*p* < 0.001), third month (*p* < 0.0001)) for serum insulin levels. **m** Significant difference between the D-insulin and D-TD groups (first and second months (*p* < 0.05)) for serum insulin levels. **t** Significant difference between the D-TD and D-insulin+TD groups (first month: (*p* < 0.01)) for serum insulin levels.

Serum insulin and glucagon levels were measured monthly for 14 weeks of treatment in all groups. After induction of diabetes, serum insulin levels in the DC group significantly decreased over three months (*p* < 0.001; Fig. 5b). Also, the serum insulin level in the DC group was significantly reduced in comparison to the NDC group (first month: (*p* < 0.01), second month (*p* < 0.001), third month (*p* < 0.0001); Fig. 5b). In the treatment groups, serum insulin levels significantly increased in comparison with DC animals during the three months (first month: (*p* < 0.01), second month (*p* < 0.001), third month (*p* < 0.0001); Fig. 5b). The highest serum insulin level was observed in the D-insulin group in comparison to D-TD (*p* < 0.05) and D-insulin+TD groups in the third month (Fig. 5b).

Serum glucagon levels were also measured monthly and the results showed that serum glucagon levels in the DC animals significantly increased in compared to the NDC group (first month: (*p* < 0.01), second month (*p* < 0.001), third month (*p* < 0.0001), Fig. 5a). The treatment groups had a significant decrease in serum glucagon levels compared to the DC group during the three months (Fig. 5a). The D-TD group showed a more effective reduction in serum glucagon levels every three months than the other two treatments (D-insulin and D-insulin +TD) groups (Fig. 5a). In the third month, the decrease in serum glucagon level in the treatment groups (*p* < 0.0001) was more than the DC group in other treatment months.

### *Glut2*, *Pdx1* mRNA gene expressions

**Fig. 6:**
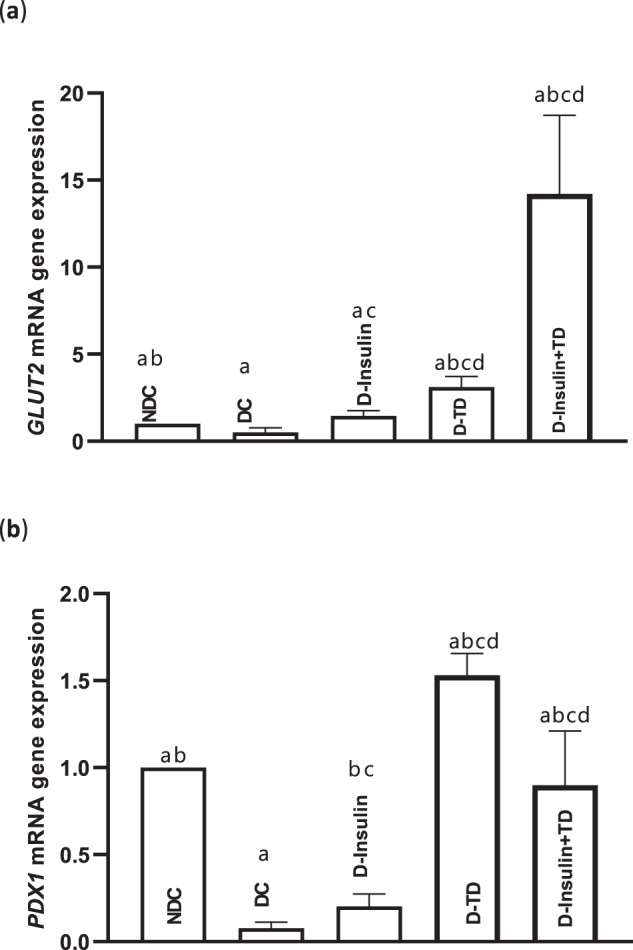
Effect of TD on mRNA expression of pancreatic genes. Comparison of mRNA expression of *Glut2* (**a**), mRNA expression of *Pdx1* (**b**) in the non-diabetic control (NDC), diabetic control (DC), D-insulin, D-TD, and D-insulin+TD groups. DC group received 60 mg/kg STZ. D-insulin group diabetic animals treated with 2.5 U/kg insulin twice per day (1/3 morning and 2/3 in the evening), D-TD group diabetic animals treated with daily IP injection of 40 mg/kg thiamine disulfide, D-insulin+TD group diabetic animals treated with TD and insulin (2.5 U/kg insulin +40 mg/kg TD once per day). Data are expressed as mean ± S.E.M (*N* = 7). **a** Significant difference in mRNA expression of *Pdx1*between the DC group and other groups (DC vs NDC (*p* < 0.05), DC vs D-insulin (*p* < 0.01), DC vs D-insulin+TD (*p* < 0.0001), DC vs D-TD (*p* < 0.001)) and significant difference in mRNA expression of *Glut2* (DC vs NDC (*p* < 0.001), DC vs D-TD (*p* < 0.0001)). **b** Significant difference in mRNA expression of *Pdx1* between the NDC group and other groups, (NDC vs D-insulin+TD (*p* < 0.001), NDC vs D-TD (*p* < 0.01)) and (NDC vs D-insulin and significant difference in mRNA expression of *Glut2* (D-TD (*p* < 0.001), and NDC vs D-insulin+TD (*p* < 0.05). **c** Significant difference between the D-insulin group and the other two treatments (D-TD and D-insulin+TD) groups (D-insulin vs D-TD (*p* < 0.01), D-insulin vs D-insulin+TD (*p* < 0.0001)) for mRNA expression of *Pdx1* and (*p* < 0.01) for mRNA expression of *Glut2*. **d** Significant difference between the D-TD and D-insulin+TD groups, (*p* < 0.05) for mRNA expression of *Pdx1* and (*p* < 0.001) for mRNA expression of *Glut2*.

There was a significant decrease in *Pdx1* gene expression in the DC group compared to the NDC group (*p* < 0.01, Fig. 6b). The expression of the *Pdx1* gene in all treatment groups significantly increased compared to the DC (*p* < 0.01) and NDC (*p* < 0.001) groups. The best expression of the *Pdx1* gene was also observed in the D-TD group (Fig. 6b). In the DC group, *Glut2* gene expression was significantly decreased compared to the NDC group (*p* < 0.01, Fig. 6a). The *Glut2* gene expression, in all treatment groups, was significantly increased in comparison to the DC group (*p* < 0.01, Fig. 6a), and in the D-insulin +TD group was higher than in other treatment groups (D-TD and D-insulin) (*p* < 0.01; Fig. 6a).

## Discussion

This study aimed to evaluate the effect of TD on improving blood glucose levels and increasing insulin sensitivity in the T1D animal model. Herein, pancreatic function and insulin sensitivity in STZ-induced diabetic rats were evaluated by applying the hyperglycemic-euinsulinemic clamp technique. Moreover, the expression of *Glut2* and *Pdx1* genes was studied. Our results revealed that administration of TD in STZ-induced diabetic rats could significantly reduce blood glucose levels and insulin resistance after 14 weeks in comparison with those of the DC group. In addition, serum levels of insulin and glucagon and expression of pancreatic genes (*Glut2, Pdx1*) showed a significant increase compared to the DC group. Furthermore, the administration of TD had a positive effect on insulin and glucagon tolerance test 14 weeks following the treatment. In our study, all the rats were monitored daily for any signs of diabetes after STZ injection, including high blood glucose levels and weight loss. Animal body weight and mean blood glucose level in the D-TD group showed a statistical difference with those of the D-insulin group.

Thiamine or vitamin B1 is a coenzyme involved in the metabolism of sugars, which is reduced in diabetes. Thiamine deficiency can exacerbate the side effects of diabetes. In thiamine deficiency, glucose is metabolized through metabolic pathways that can stimulate insulin resistance and the complications of diabetes [19]. Thiamine maintains carbohydrate metabolism by participating in several cellular metabolic processes [37]. In addition, it prevents the formation of AGEs in hyperglycemic conditions [38]. In the STZ-induced diabetic rats, the effect of a high dose of thiamine or benfotiamine (a lipophilic form of thiamine) was previously reported on the reduction in plasma’s AGEs in [39].

We showed that following the induction of diabetes, the area under the glycemic curve of ITT compared to the NDC group, increased while the response of insulin target cells to exogenous insulin decreased [35]. The hyperglycemic-euinsulinemic clamp technique demonstrated a decline in the sensitivity of insulin target cells to insulin. A comparison of all the treatment groups implied that the protocol performed in the D-TD group was more effective in blood glucose and AUC than that in D-insulin and D-insulin + TD groups. Previous studies have shown that all the pathological processes observed in the brain during thiamine deficiency are strongly associated with the pathophysiology of insulin resistance and macrovascular disease; yet, thiamine supplementation can ameliorate all these complications [40, 41]. Thiamine deficiency impairs the synthesis and secretion of insulin due to decreased glucose oxidation; on the other hand, insulin deficiency can aggravate thiamine deficiency [42], which is also strongly associated with pathophysiological resistance in the body [43].

The results of GTT indicated that after glucagon injection, blood glucose levels in the DC group significantly rose compared to those in the NDC group. The area under the glycemic curve also decreased in all the treatment groups compared to that of the DC group. Administration of TD improved the blood glucose level in the D-TD and D-insulin+TD groups. Moreover, this amended the GTT’s result in the D-TD group, suggesting that the pancreatic β-cells in this group can secret insulin. In the DC group, IP injection of glucagon raised blood glucose level, but could not return to its original state after 2 hours due to the inability to secrete insulin. In all the treatment groups, 30 minutes after glucagon administration, the blood glucose level significantly decreased compared to that of the DC group; it is probably on account of the promoted function of the pancreas to secrete insulin in these groups. Glucagon is involved in the hepatic gluconeogenesis pathway and can increase hyperglycemia. Glucagon increases hepatic glucose output through the gluconeogenesis pathway [44] while this pathway is suppressed by insulin; thus, hepatic glucose output will decrease [45]. The reason why the blood glucose level in the D-TD group was lower than that in the DC group was probably the inhibition of gluconeogenesis enzymes. Conceivably, TD could improve GTT; accordingly, glucagon prevented the overactivity of the glycogenolysis pathway and incomplete carbohydrate metabolism. Therefore, the improvement of hyperglycemia in the D-TD group reduced the effect of glucagon on hepatic glucose production.

The results of IIR showed that insulin sensitivity increased in the DC group compared to that in the NDC group. However, in the treated groups, the sensitivity to insulin response increased compared to the DC group; insulin sensitivity in the D-TD group was significantly higher than that in the D-insulin group. Insulin therapy in T1D can reduce insulin resistance and promote β-cells function by lowering blood glucose levels. Euglycemic-hyperinsulinemic research on T1D patients has suggested that insulin sensitivity decreased in these patients and that there was a relationship between insulin sensitivity, insulin dose, and HbA1c. A study reported that insulin-mediated glucose excretion is reduced in both euglycemic and hyperglycemic insulin clamps in T1D patients [46, 47]. There are several hypotheses to explain the decrease in insulin sensitivity in T1D, including prolonged exposure to supraphysiological levels of exogenous insulin, genetic factors, failure to deliver insulin into the bloodstream, decreased insulin delivery to the liver, decreased hepatic IGF-1 production, abnormal regulation of glucagon, fatty acid exposure, and lipid toxicity (NEFA) [9, 48]. It has also been reported that thiamine deficiency is higher in T1D than in T2D; hence, thiamine deficiency has been suggested as a mediator of insulin resistance in diabetes [43, 49]. Insulin plays a pivotal role in the insulin sensitivity of target tissues. Thiamine is essential for insulin synthesis and secretion; thiamine deficiency in diabetic conditions affects insulin synthesis and secretion, serum insulin levels, and glucose transporters. All the above-mentioned procedures lead to metabolic dysfunction in hyperglycemic conditions and decreased insulin sensitivity [21, 50]. Hence, thiamine supplements in diabetic patients during hyperglycemia could advance insulin function. The striking reduction in insulin-mediated glucose uptake can infer hyperinsulinemia, which in turn increases free radical production. Herein, an improvement was observed in the D-TD group concerning glucose metabolism and insulin function. It could be thus concluded that TD affects the maintenance of β-cells activity by reducing oxidative stress.

We assessed insulin resistance via ITT index; the obtained findings represented a significant decrease in blood glucose (20 min. after insulin administration) in all the treatment groups compared to the DC group. This response was better in the D-TD group owing to a decrease in insulin resistance. After 14 weeks, a decreased insulin level and an increased glucagon level were observed in the DC group compared to the NDC group. However, in the D-TD and D-insulin groups, insulin levels significantly increased whereas glucagon levels significantly decreased. Circulating insulin affected glucagon function; at high insulin levels, the effect of glucagon on the liver declined, resulting in lower blood glucose levels [51, 52].

Numerous studies have shown that thiamine deficiency in diabetic rats reduces glucose oxidation and insulin secretion [53], which is modified by thiamine administration. Other papers have suggested that high doses of thiamine may reduce the need for exogenous insulin [54]. We found that the administration of TD positively affected glucose metabolism and insulin secretion. TD improved blood glucose level and insulin function in diabetic rats; accordingly, TD activates glucose metabolism and insulin synthesis preventing glucose intoxication due to hyperglycemia in TDM.

According to our results regarding the ITT and the GTT, it seems as if TD can repair damaged pancreatic β-cells and increase insulin secretion. Furthermore, TD may reduce insulin resistance and increase insulin sensitivity by improving pancreatic β-cells function, increasing insulin secretion, and decreasing glucagon levels. Thus, TD (a lipophilic form of vitamin B1) can improve hyperglycemia, which contributes to increased endogenous insulin secretion and decreased glucagon secretion.

We observed an increase in *Pdx1* and *Glut2* gene expression in the D-TD and D-insulin groups compared to the DC group, which leads to ameliorated glucose tolerance and prominent insulin secretion by the pancreas.

In conclusion, TD could play an effective role in improving hyperglycemia in T1D rats [16, 55]. TD may affect insulin and glucagon secretion by increasing the expression of genes involved in pancreatic insulin secretion. In this regard, previous research has shown that *Pdx1* and *Glut2* nuclear transmission is impaired in high-fat diabetic rats [56, 57]. TD seems to be able to improve the function of pancreatic β-cells in insulin secretion by affecting *Pdx1* and *Glut2* genes expression. Although insulin therapy in diabetic animals increases the expression of these genes, it is not as effective as thiamine. The regulation effect of TD on glucose metabolism may be mediated by modifying the expression of the β-cells genome in order to increase insulin secretion, elicit insulin responses at the insulin target cells, and increase insulin sensitivity. According to our findings, administration of TD, as a lipophilic thiamine supplement, had interaction effects on the improvement of STZ-induced hyperglycemia. Thus, in addition to exogenous insulin, prescribing the TD, as a natural supplement, contributes to the amelioration of diabetic patients.

## Conclusion

In the current work, we showed that TD injection improved hyperglycemia in male type 1 diabetes rats. Administration of TD had a positive effect on serum insulin and glucagon concentrations in diabetic rats by increasing serum insulin levels, decreasing serum glucagon levels, enhancing insulin sensitivity, promoting the pancreas function and pancreatic cells survival via increased *Glut2* and *Pdx1* genes expression, and diminishing the dose of insulin exogenous.

## Data Availability

The data of the present study is available in the endocrine and metabolism lab in the physiology department
